# Cervico-shoulder dystonia following lateral medullary infarction: a case report and review of the literature

**DOI:** 10.1186/s13256-018-1561-y

**Published:** 2018-02-10

**Authors:** Takashi Ogawa, Yuri Shojima, Takuma Kuroki, Hiroto Eguchi, Nobutaka Hattori, Hideto Miwa

**Affiliations:** 1 Department of Neurology, Juntendo University Nerima Hospital, 3-1-10 Takanodai, Nerima, Tokyo, 177-8521 Japan; 2 Department of Neurology, Juntendo University School of Medicine, 1-21-1 Hongo, Bunkyo, Tokyo, 113-0033 Japan

**Keywords:** Cervical dystonia, Lateral medullary infarction, Opalski’s syndrome, Literature review, Case report

## Abstract

**Background:**

Secondary cervical dystonia is induced by organic brain lesions involving the basal ganglia, thalamus, cerebellum, and brain stem. It is extremely rare to see cervical dystonia induced by a medullary lesion.

**Case presentation:**

We report a case of an 86-year-old Japanese woman who developed cervical dystonia following lateral medullary infarction. She developed sudden-onset left upper and lower extremity weakness, right-side numbness, and dysarthria. Brain magnetic resonance imaging revealed an acute ischemic lesion involving the left lateral and dorsal medullae. A few days after her stroke, she complained of a taut sensation in her left neck and body, and cervico-shoulder dystonia toward the contralateral side subsequently appeared. Within a few weeks, it disappeared spontaneously, but her hemiplegia remained residual.

**Conclusions:**

To date, to the best of our knowledge, there has been only one reported case of cervical dystonia associated with a single medullary lesion. It is interesting to note the similarities in the clinical characteristics of the previously reported case and our patient: the involvement of the dorsal and caudal parts of the medullary and associated ipsilateral hemiplegia. The present case may support the speculation that the lateral and caudal regions of the medulla may be the anatomical sites responsible for inducing cervical dystonia.

## Background

Cervical dystonia is a focal dystonia characterized by sustained, involuntary contraction of the neck muscles, resulting in abnormal movements and postures of the head [1]. It is known that cervical dystonia is induced by focal organic lesions involving various regions of the brain. Primarily caused by lesions in the cerebrum, including the caudate nucleus, putamen, pallidum, thalamus, frontal cortex, and parietal cortex, lesions in the cerebellum and/or brain stem are also able to cause secondary cervical dystonia [2]. Recently, we encountered a patient who developed cervico-shoulder dystonia following lateral medullary infarction. We present the clinical data of the patient and review cases of other patients with secondary cervical dystonia caused by brain stem lesions.

## Case presentation

An 86-year-old Japanese woman was admitted to our hospital with the sudden appearance of weakness in the left upper and lower extremities, numbness of the right upper and lower extremities, and dysarthria. Her family history was unremarkable. Particularly, she had no family history of movement disorders. She had hypertension, diabetes mellitus, and dyslipidemia. At age 82, she had developed hemiparesis caused by a lacunar infarction of the left capsulothalamic region, and her neurological symptoms fully improved without sequelae.

On admission, her blood pressure was 202/98 mmHg, but her heartbeat was regular. Her other general status was unremarkable. A neurological examination revealed that she was alert and oriented, without dementia. Her cranial nerves were intact, but her speech was mildly dysarthric. Horner’s sign was not noted. Hemiparesis was assessed according to the Medical Research Council scale and was noted in the upper and lower extremities with manual muscle strength scale scores of 3 and 4 for the upper and lower extremities, respectively. No pathological reflex was noted. Her superficial sensation was disturbed in her upper and lower extremities and body on the right side. Her position sensation was disturbed in her left upper and lower extremities. Her vibration sensation was intact. Her National Institutes of Health Stroke Scale assessment yielded 3 points.

**Fig. 1:**
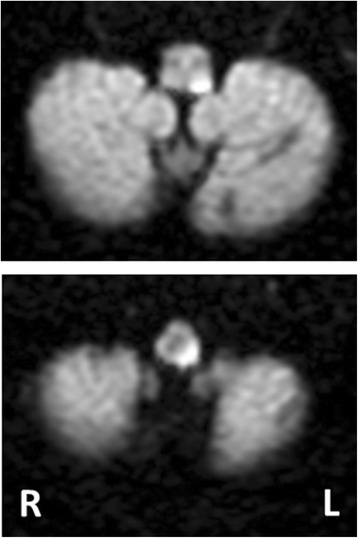
Brain magnetic resonance imaging of axial diffusion-weighted imaging demonstrating a high signal intensity lesion involving the lateral and dorsal medullae

**Fig. 2:**
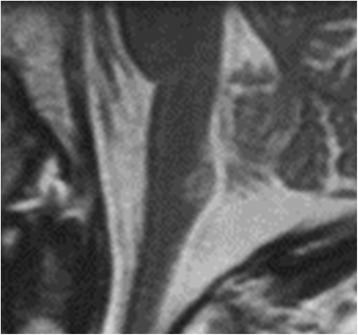
Sagittal T2-weighted imaging revealing a lesion involving the caudal medulla

**Fig. 3:**
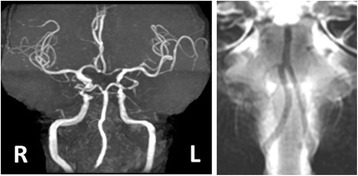
Magnetic resonance angiography revealing the disappearance of the left vertebral artery (left panel). Basi-parallel anatomic scanning magnetic resonance imaging delineates the outside shape of the left vertebral artery, indicating that the vertebral artery was not hypoplastic but obstructed (right panel)

Brain magnetic resonance imaging (MRI) at admission (day 1) did not reveal ischemic lesions. However, brain MRI performed again at day 2 did reveal an ischemic lesion in the left lateral lower medulla (Figs. 1 and 2). Magnetic resonance angiography showed a decreased left vertebral artery signal (Fig. 3a). Basi-parallel anatomic scanning (BPAS) MRI delineated the outside shape of the left vertebral artery (Fig. 3b), suggesting that the vertebral artery might be obstructed. This was confirmed by 3D computed tomographic angiography.

**Fig. 4:**
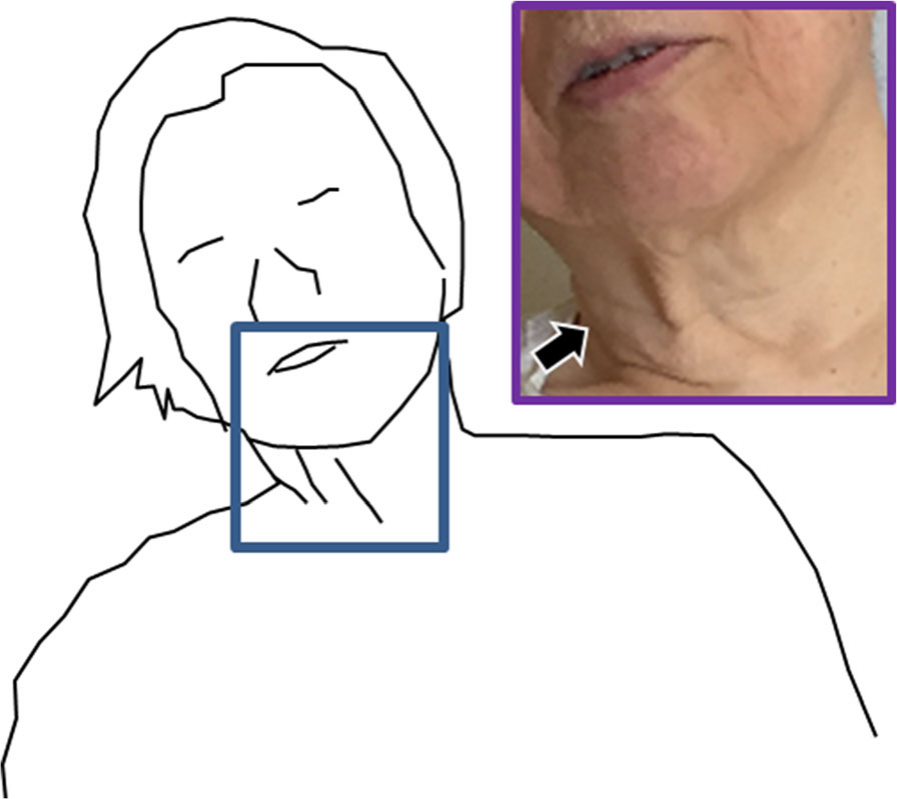
Cervico-shoulder dystonia of our patient. The head and trunk are leaning to the right side, with contraction of the right lateral neck muscles, including the right sternocleidomastoid muscle (*arrow*)

A few days after admission, the patient experienced sustained pain and a taut sensation, localized in her left neck. Several days afterward, her head was found involuntarily deviated to the right, with tonic contraction of her right sternocleidomastoid and trapezius muscles (Fig. 4). On occasion, her left shoulder and arm elevated involuntary. We suspected that she had cervico-shoulder dystonia. Within a few weeks, her dystonic symptoms as well as her neck pain gradually improved and disappeared, although she had residual neurological sequelae, such as left hemiparesis (ipsilateral hemiplegia, called *Opalski’s syndrome*), right hemisuperficial sensory loss, and disturbance of the left position sense.

## Discussion

Our patient experienced secondary cervico-shoulder dystonia following acute lateral medullary infarction. In addition, the lateral medullary lesion did not induce Wallenberg syndrome, but it did produce Opalski’s syndrome.

Opalski’s syndrome is a classically known syndrome, a rare variant of lateral medullary syndrome characterized by ipsilateral hemiplegia. Lesions responsible for Opalski’s syndrome are usually located within the sub-bulbar part of the medulla. From an anatomical perspective, it is presumed that the ipsilateral hemiplegia caused is due to the involvement of the lateral corticospinal tract after pyramidal decussation [3]. Also, in our patient, the lesion was localized in the tegmentum of the caudal medulla, potentially disrupting the ipsilateral lateral corticospinal tract (Figs. 1 and 2).

A comprehensive review of the literature has been published regarding cerebellar lesion-induced cervical dystonia [4]. However, no review of the relationship between brain stem lesions and secondary cervical dystonia has been published to date. Therefore, we review the literature on secondary cervical dystonia due to brain stem lesions (Table 1) [5–13]. To the best of our knowledge, between 1979 and 2016, a total of 18 cases with secondary cervical dystonia have been reported, including our patient. The age of onset varies from 15 months to 86 years. The phenotypes of dystonia are heterogeneous, and various head positions can be induced, such as rotation, laterocollis, anterocollis, or retrocollis, with or without shoulder elevation. Of note, of 18 patients, 13 (72%) presented with rotation of the head, 12 (67%) had laterocollis, and 7 (54%) had both rotation and laterocollis. The relationship between the lesion side and the direction of torticollis is 25% ipsilateral and 58.3% contralateral. A single brain stem lesion can be sufficient to induce cervical dystonia because multiple brain stem lesions were observed in only four patients. Various background disorders are responsible for secondary cervical dystonia: cerebrovascular stroke (*n* = 8 [44.4%], including four infarction cases and four hemorrhage cases), brain tumor (*n* = 8 [44.4%], including two schwannoma cases and one case each of astrocytoma suspected, meningioma, ependymoma, gangliocytoma, arachnoid cyst, cavernous, and hemangioma), multiple sclerosis (*n* = 1), and diffuse axonal injury (*n* = 1). This suggests that stroke and tumor are the most common etiologies. Outcomes are variable, but spontaneous improvement is reported in almost half of the cases. As shown in Table 1, the clinical characteristics of the cases are heterogeneous, so no overt relationship between the lesion location and cervical dystonia seems to exist.

**Table 1:** Review of brain stem lesions causing secondary cervical dystonia

Case report number	First author, year [reference]	Age, sex	Dystonia features	Brain lesion	Cause or pathogenesis of lesion	Other clinical features noted	Onset from diagnosis	Treatment for dystonia	Cervical dystonia outcome
1	Boisen, 1979 [5]	32 years, F	Right rotation	Midline between cerebellar tonsils and medullaris	Ependymoma	None	NA	NA	NA
2	Plant *et al*., 1989 [6]	30 years, F	Left rotation	Large lesion: right mesencephalon to lower edge of thalamus Small lesion: right cerebellar hemisphere	Multiple sclerosis	Gait ataxia Left hemisensory disturbance (pain, temperature)	1 year	None	Persisted at 1 year
3	Krauss *et al*., 1992 [7]	4 years, M	Right laterocollis Left rotation	Diffuse lesion: paramedian and lateral pontomesencephalic tegmentum mid-pons thalamus	Diffuse axonal injury	Facial palsy Right hemidystonia Ipsilateral hemiparesis Right intention tremor	6 months	Thalamotomy	Marked improvement
4	Caress *et al*., 1996 [8]	4 years, M	Left rotation	Right cerebellum Right medulla Right pons Cervical spinal cord	Cerebellar gangliocytoma	Gait ataxia	NA	Subtotal resection	Improvement
5	Krauss *et al*., 1997 [9]	42 years, M	Left laterocollis	Left cerebellopontine angle	Schwannomas	Left progressive hearing loss Head jerking toward the left Shrugging of the left shoulder	NA	Operation botulinum toxin	Once mild improved, but with recurrenceand did not improve on any medication but botulinum toxin
6		13 years, F	Right rotation	Left cerebellopontine angle	Schwannomas	Cerebellar ataxia Decreased hand dexterity Slightly slurred speech Mild left spinal accessory nerve palsy	NA	Shunting procedure for obstructive hydrocephalus	Relieved after 1 year
7		52 years, F	Left laterocollis Left rotation	Left cerebellopontine angle	Meningioma	Head horizontal oscillation toward the left	6 years	Botulinum toxin Trihexyphenidyl Cyclobenzaprine primidone Amitriptyline Carbamazepine	Improved but limited effect
8	LeDoux *et al*., 2003 [2]	55 years, M	Left rotation Abnormal contraction of right sternocleidomastoid muscle	Right central pons	Spontaneous hemorrhage	Left hemiparesis Dysarthria Bilateral abducens palsy	24 hours	None	4–6 weeks after hypertrophy of the right sternocleidomastoid muscle
9		42 years, F	Left rotation Right laterocollis Right shoulder elevation (mild)	Left cerebellopontine angle	Arachnoid cyst	None	At diagnosis	Botulinum toxin medication	Did not improve
10		67 years, F	Right laterocollis (severe) Anterocollis Right shoulder moderate elevation and anterior displacement Left rotation (mild)	Multiple lesions in pons and caudal midbrain	Ischemic infarctions	Dysarthria Hyperreflexia Impaired conjugate Mild right hemiparesis Right hand parkinsonian-type resting tremor Right arm action tremor Spastic and ataxic gait	Several days	Levodopa/carbidopa Botulinum toxin	Did not improve with levodopa Moderately improved by botulinum toxin
11		72 years, M	Left rotation Retrocollis	Central pons Left posterior thalamus Left occipital lobe	Multiple infarction	Right homonymous hemianopsia Anomia Right hemihypesthesia	1 day	NA	NA because of death
12	Kajimoto *et al*., 2004 [10]	84 years, F	Right laterocollis	Left lateral caudal medulla	Ischemic infarction	Left hemiparesis Left body sensory disturbance (pain, touch, temperature) Left paretic sternocleidomastoid muscle Left decreased deep sensation Left neck pain	10 days	None	Gradually improved after several weeks
13	Loher *et al*., 2009 [11]	31 years, M	Right laterocollis Left rotation	Tegmental and tectal pons Right mesencephalon	Spontaneous hemorrhage	Right sixth and seventh nerve palsies Left hemidystonia and athetoid movements Orofacial dystonia Right head jerky tremor	3 months	Propranolol l-Tryptophan l-Hydroxytryptophan Trihexyphenidyl	Did not improve
14		42 years, M	Right laterocollis Left rotation	Left dorsolateral pons Left middle cerebellar peduncle	Posttraumatic hemorrhage	Oculomotor disturbances Dysarthria Flaccid tetraparesis and ataxia Left hemidystonia	14 months	None	Did not improve
15		56 years, M	Right laterocollis Left rotation	Left dorsolateral pons Left middle cerebellar peduncle	Spontaneous hemorrhage	Fifth and seventh nerve palsies Gaze palsy (upward and horizontal) Left hemidystonia Blepharospasm	1 month	None	Gradually improved
16	Agrawal *et al*., 2009 [12]	9 years, F	Right laterocollis	Left midbrain and pons	Cavernous hemangioma hemorrhage	Left cerebellar signs	At diagnosis	Left retromastoid Suboccipital craniotomy	Significant improvement at 8 months
17	DeBenedictis *et al*., 2010 [13]	15 months, F	Left laterocollis	Left brachium pontis (displacement of pons)	Tumor (low-grade astrocytoma suspected)	Left eye tearing Extreme photophobia Epiphora	At diagnosis	None	Resolved in 1 year
18	Our patient	86 years, F	Right laterocollis Left shoulder elevation	Left dorsal lower lateral medulla	Ischemic infarction	Left hemiplegia Dysarthria Right body sensory disturbance Left athetoid movement	A few days	None	Spontaneous improvement in a few weeks

*NA* Not available

However, of these patients with brain stem lesion-induced cervical dystonia, only one case had a single medullary lesion [10]. It is interesting to note that that case exhibited clinical similarities to our patient: the appearance of cervical dystonia toward the contralateral side following the stroke, which disappeared spontaneously; the dorsal, lateral, and caudal parts of the medulla were involved; and with an association with ipsilateral hemiplegia (Opalski’s syndrome). In both cases, it can be speculated that the lesions affected the afferent fibers of the cerebellum with the lateral corticospinal tract. However, it remains unclear whether this type of cervical dystonia may be induced following interruption of the spinocerebellar tract at the caudal medulla. Further accumulation of similar cases is required to better understand the pathophysiological mechanisms underlying secondary cervical dystonia.

One problem that should be considered is whether malfunctioning of the vestibular system possibly influences the head and/or truncal position in these patients with medullary infarction. Indeed, head leaning and body lateropulsion are known to occasionally be induced by a lateral medullary lesion, wherein the vestibular nucleus is located. However, we suspect that impairment of the vestibular system was not related to the mechanism underlying cervical dystonia in our patient or in the similar previously reported case, because the direction of head leaning was ipsilateral to the medullary lesion [14], whereas the direction of cervical dystonia in our patient was contralateral. In addition, the location of the medullary infarction in our patient was clearly caudal to the level wherein the vestibular nucleus is located.

## Conclusions

Although it remains uncertain whether cervical dystonia is more likely to be complicated by Opalski’s syndrome, this type of cervical dystonia might be overlooked in patients with acute stroke, particularly those with hemiparesis. We believe this case report contributes to recognizing the possible relationship between caudal medullary lesions and cervical dystonia, as well as facilitates the accumulation of similar cases for better understanding of secondary dystonia.

## Abbreviations

BPAS: Basi-parallel anatomic scanning
MRI: Magnetic resonance imaging
